# Sarcome d'Ewing extrasquelettique de l'espace parapharyngé à propos d'un cas

**DOI:** 10.11604/pamj.2024.48.103.44109

**Published:** 2024-07-12

**Authors:** Fatine Aboutajdine, Lyazid Maskani Filali, Ahmed Sqalli Houssini, Zainab Hayat, Mouna Ouazzani Touhami, Abdeljali El Quessar, Hassan El Edghiri

**Affiliations:** 1 Service d'Otorhinolaryngologie et de Chirurgie Cervico-Faciale, Hôpital Universitaire International Cheikh Zaid, Université des Sciences de la Santé, Rabat, Maroc,; 2 Service de Radiologie, Hôpital Universitaire International Cheikh Zaid, Université des Sciences de la Santé, Rabat, Maroc

**Keywords:** Sarcome d'Ewing, extrasquelettique, espace parapharyngé, ORL, cas clinique, Ewing's sarcoma, extraskeletal, parapharyngeal space, ENT, case report

## Abstract

Le sarcome d'Ewing extrasquelettique est une tumeur maligne le plus souvent retrouvée chez les enfants et les adolescents dont la localisation cervico-faciale reste rare. Dans l'espace parapharyngé, le diagnostic est souvent tardif avec un pronostic péjoratif. Nous rapportons le cas clinique d'une patiente âgée de 37 ans, admise en urgence pour dyspnée inspiratoire dans un tableau d'asthénie et d'anorexie. L'imagerie par résonance magnétique (IRM) cervicale a objectivé un processus au dépend de l'espace parapharyngé droit obstruant la quasi-totalité de la lumière pharyngée. L'étude anatomopathologique, immunohistochimique et cytogénétique, a conclu à un sarcome d'Ewing extrasquelettique. Le bilan d'extension était négatif. La patiente a reçu de la chimiothérapie. Le sarcome d'Ewing extrasquelettique de l'espace parapharyngé est une entité très rare: seulement 4 cas ont été retrouvés dans la littérature. Néanmoins les caractéristiques cliniques et paracliniques restent semblables. C'est un néoplasie qui doit être connue afin de porter un diagnostic précoce en vue d'améliorer le pronostic et la prise en charge

## Abstract (en)

Extraskeletal Ewing's sarcoma is a malignant tumour most often found in children and adolescents, but rarely in the cervicofacial region. When it affects the parapharyngeal space, diagnosis is often delayed, resulting in poor prognosis. We here report the clinical case of a 37-year-old female patient admitted to the emergency department with inspiratory dyspnea, asthenia and anorexia. Cervical spine MRI revealed a process in the right parapharyngeal space obstructing almost the entire pharyngeal lumen. Anatomopathological, immunohistochemical and cytogenetic examinations showed extraskeletal Ewing's sarcoma. The metastatic workup was negative. The patient received chemotherapy. Extraskeletal Ewing's sarcoma of the parapharyngeal space is extremely rare: only four cases have been reported in the literature. Nonetheless, clinical and paraclinical characteristics are similar. It is a neoplasm that must be recognized for early diagnosis to improve prognosis and management.

## Introduction

La famille des tumeurs neuroectodermiques primitives périphériques (pPNET) regroupe des tumeurs osseuses et des tissus mous atteignant principalement les enfants et les adolescents, et plus rarement l'adulte. Le sarcome d'Ewing extra-squelettique (SEE) a été décrit pour la première fois en 1969 par Tefft *et al*. [1]. Il s'agit d'une tumeur maligne rare, caractérisée histologiquement par des cellules rondes basophiles avec une positivité membranaire du CD99 à l'immunohistochimie semblable au sarcome d'Ewing osseux qui dérive du tissu mésenchymateux. Ses sites de prédilections sont le tronc, les extrémités et l'abdomen. Les localisations cervicale et faciale sont exceptionnelles. L'étude immunohistochimique et cytogénétique sont toujours de mise et essentielles au diagnostic. L'association chimiothérapie et chirurgie avec des marges de résection larges et/ou radiothérapie améliore le pronostic de survie à 5 ans.

## Patient et observation

**Présentation du patient**: il s'agit d'une patiente de 37 ans qui a consulté pour une dysphagie mixte d'installation progressive dans un contexte d'amaigrissement et d'altération de l'état général. Les antécédents sont sans particularités. L'évolution a été marquée par une dyspnée inspiratoire d'aggravation progressive. La patiente s'est présentée en dyspnée laryngée ce qui a conduit à une trachéotomie en urgence.

**Figure 1:**
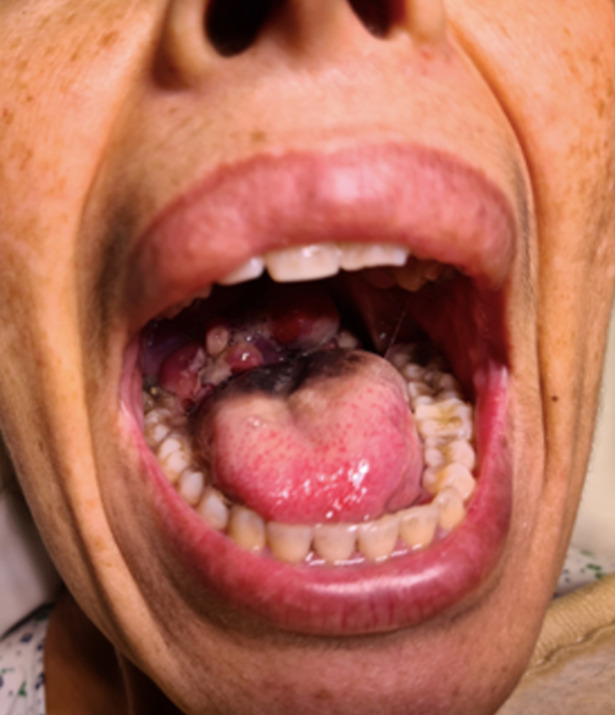
examen de la cavité buccale mettant en évidence une masse comblant la lumière pharyngée

**Résultats cliniques**: l'examen clinique général retrouve une patiente bien orientée dans le temps et l'espace, avec état hémodynamique stable. L'examen ORL a mis en évidence une masse parapharyngée droite comblant quasi-totalement la lumière pharyngée et obstruant les voies aériennes supérieures (Figure 1).

**Chronologie**: après l'examen clinique, la patiente a bénéficié d'une IRM cervicale puis d'une biopsie de la masse parapharyngée sous anesthésie locale dont l'examen anatomopathologique a motivé la réalisation d'un bilan d'extension à base de tomodensitométrie (TDM) thoraco-abdomino-pelvienne et de scintigraphie osseuse.

**Figure 2:**
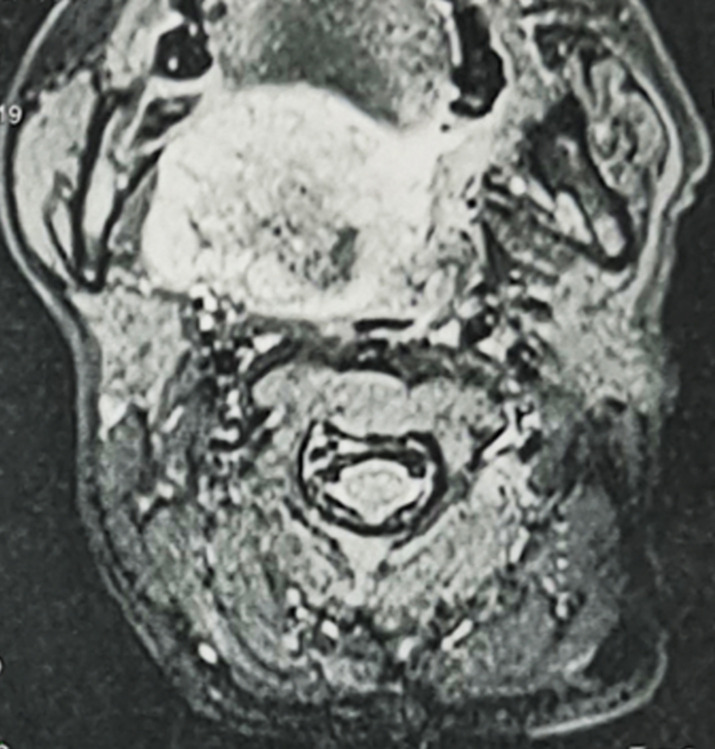
IRM cervicale en coupe axiale en séquence T2

**Figure 3:**
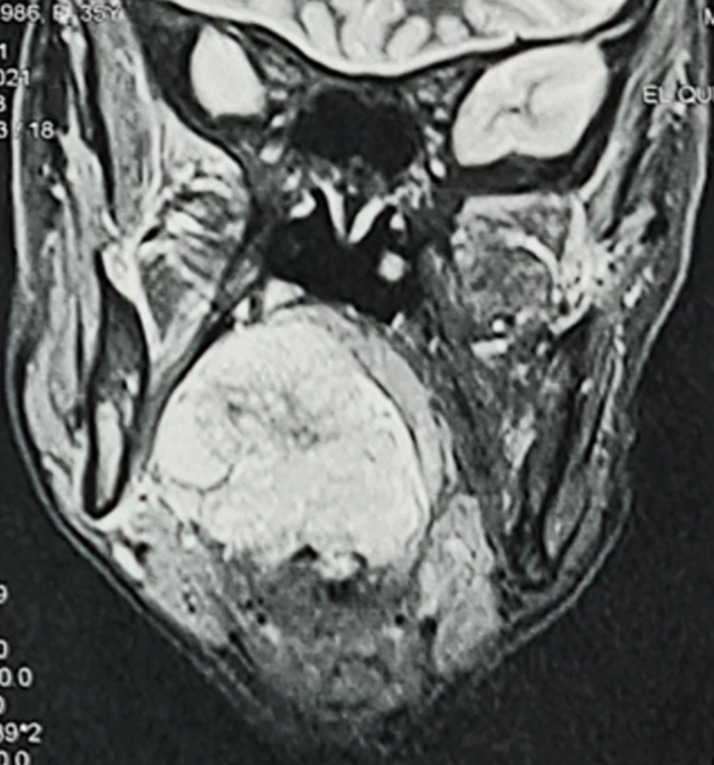
IRM cervicale en coupe coronale en séquence T2

**Démarche diagnostique**: l'IRM cervicale a montré un processus tumoral de l'espace parapharyngé droit bombant dans le pharynx en hypersignal T2 avec restriction en diffusion et se rehaussant après injection de gadolinium. Il mesure 47 x 40 x 50 mm. La tumeur occupe la quasi-totalité de la lumière pharyngée et envahit le voile du palais en haut. Cette dernière est bourgeonnante contenant des zones de nécrose par endroits (Figure 2, Figure 3). Une biopsie de la masse avec examen anatomopathologique a mis en évidence des cellules rondes tumorales basophiles. L'étude immuno-histochimique était positive pour les protéines CD99, anticorps anti-S100 et Ki67. De plus, une étude cytogénétique a été réalisée sur le prélèvement à la recherche d'une translocation EWS/FLI1 dont le résultat était positif. Le résultat final anatomopathologique a conclu à un sarcome d'Ewing extraosseux de l'espace parapharyngé. Le bilan d'extension était négatif.

**Intervention thérapeutique**: la patiente a été référée en oncologie pour chimiothérapie première à base de vincristinne, ifosfamide, doxorubicine et etoposide.

**Suivi et résultats des interventions thérapeutiques**: après 6 cures de chimiothérapie, la progression de la tumeur a continué et la patiente est décédée dû à l'extension basicranienne de la maladie.

**Consentement éclairé**: la patiente a donné son consentement pour la publication de son cas.

## Discussion

Le sarcome d'Ewing extrasquelettique de l'espace parapharyngé est une entité rare. Seuls quatre cas ont été rapportés dans la littérature (PubMed) dont seulement deux chez l'adulte. Le premier cas a été publié en 2004 par Ng *et al*. à propos d'un patient âgé de 53 ans ayant une tumeur de l'espace parapharyngé droit avec envahissement de la base du crâne et extension intracrânienne. Le patient est décédé 6 mois après son diagnostic [2]. Le deuxième cas a été décrit en 2009 par Chaudhary *et al*. à propos d'un garçon de 6 ans avec une tumeur de l'espace parapharyngé droit avec extension intra-orbitaire et intracrânienne. Il a reçu une chimioradiothérapie et a été déclaré en rémission à 10 mois du début du traitement [3]. Le troisième cas est publié par Ramos-rivera *et al*. à propos d'une femme de 23 ans ayant une masse parapharyngée gauche qui a été traitée par chimiothérapie suivie d'une transplantation de cellules souches autologue. La patiente est décédée 14 mois plus tard mais son autopsie n'a révélé aucune tumeur résiduelle [4]. Enfin le quatrième cas est présenté par Khosla *et al*. à propos d'une fille de 8 ans présentant une tumeur de l'espace parapharyngé gauche. La patiente a reçu une chimioradiothérapie à la suite de laquelle elle est décédée dans un tableau de poursuite évolutive [5].

Cliniquement, le SEE se présente comme une masse indolore d'augmentation progressive comprimant les structures de voisinage [2-5]. Les examens radiologiques sont non spécifiques. L'IRM reste l'examen de référence. Le diagnostic de sarcome d'Ewing des parties molles doit être évoqué devant: une lésion hétérogène surtout sur les séquences en pondération T1 et T2, prenant le contraste et présentant des zones nécrotiques, mesurant plus de 5cm de diamètre, à localisation sous aponévrotique, de contours irréguliers ou lobulés avec des parois et septas intra tumoraux irréguliers et épais [6].

L'examen anatomopathologique est la clé du diagnostic. Histologiquement, ce sont des petites cellules rondes aux contours irréguliers sur un stroma richement vascularisé. L'étude immunohistochimique est positive pour le CD99, anti-S100 et Ki 67. L'étude cytogénétique, par FISH (hybridation in situ en fluorescence) ou par RT-PCR, est plus spécifique que l'immunohistochimie. Elle recherche un transcrit de fusion EWRS1-Fli1 positif [7,8].

Le traitement repose sur la chirurgie et ou la radiothérapie couplée à la chimiothérapie. La chirurgie est essentielle et doit être réalisée avec des marges de résection larges pour prévenir toute récidive. Elle est souvent précédée par une chimiothérapie d'induction à base de Vincristine-Ifosfamide- Doxorubicine-Etoposide (VIDE, protocole eurowing). La radiothérapie peut être envisagée en complément dans deux cas figures: premièrement, si l'exérèse chirurgicale est incomplète, une radiothérapie à la dose de 44-54Gy est recommandée; deuxièmement, en cas de contre-indication à la chirurgie, une radiothérapie à la dose de 55-60GY est indiquée [9].

## Conclusion

Le sarcome d'Ewing extrasquelettique de l'espace parapharyngé chez l'adulte est peu commun. Le diagnostic positif de cette entité reste très difficile. L'étude anatomopathologique alliant histologie, immunohistochimie et cytogénétique reste le gold standard pour le diagnostic. La chimiothérapie est le traitement de première intention associée à la chirurgie lorsque les conditions le permettent car le diagnostic est souvent tardif.
